# SelectVote Byzantine Fault Tolerance for Evidence Custody: Virtual Voting Consensus with Environmental Compensation

**DOI:** 10.3390/s25226846

**Published:** 2025-11-08

**Authors:** Belinda I. Onyeashie, Petra Leimich, Sean McKeown, Gordon Russell

**Affiliations:** School of Computing, Engineering & the Built Environment, Edinburgh Napier University, Edinburgh EH10 5DT, UK; p.leimich@napier.ac.uk (P.L.); s.mckeown@napier.ac.uk (S.M.); g.russell@napier.ac.uk (G.R.)

**Keywords:** byzantine fault tolerance, directed acyclic graph, consensus mechanisms, digital evidence custody, environmental compensation, weight verification, forensic evidence management, virtual voting, distributed ledger technology, evidence chain of custody

## Abstract

Digital evidence custody requires consensus protocols that guarantee immediate and deterministic finality. Legal admissibility depends on proof that no party can alter or delay confirmation of evidence transfers. Conventional Byzantine fault tolerance protocols scale poorly because of quadratic communication overhead, while probabilistic ledger systems such as IOTA and SPECTRE produce confirmation uncertainty that weakens custody verification. This paper introduces SelectVote Byzantine Fault Tolerance, a deterministic consensus protocol that infers virtual votes from graph structure instead of exchanging explicit messages. The protocol operates in permissioned forensic networks and assigns validation witnesses through a fixed, hash-based selection process. Empirical evaluation demonstrates sub-quadratic communication scaling ($O(n^{1.7})$) compared to traditional $O(n^{2})$ Byzantine protocols and maintains Byzantine resilience. To ensure physical integrity, the paper also presents an environmental compensation framework for precision weight verification. The framework models temperature, humidity, and pressure effects on load cells and corrects measurement drift to preserve sub-gram accuracy across normal storage conditions. Experimental evaluation confirms that the integrated system sustains high throughput with deterministic finality and maintains consistent measurement precision under environmental variation. The combined result supports reliable, legally defensible custody of digital evidence across distributed institutions.

## 1. Introduction

Modern criminal investigations depend heavily on digital devices, and custody of digital evidence must resist tampering to preserve forensic validity. The UK National Police Chiefs’ Council observes that more than 90% of reported crimes now include a “digital element” [1,2]. Investigations often seize multiple devices, and those devices may hold volatile state which degrades quickly unless handled under strict control.

Tampering with hardware or internal components can corrupt digital evidence without leaving clear signs under standard monitoring procedures. For example, an adversary might replace a memory chip, insert covert extraction circuitry, or swap the battery. Such manipulations may alter device mass by fractions to tens of grams. Detecting them reliably requires measurement systems whose resolution and stability exceed these changes.

Many commercial or industrial weighing systems operate with tolerances of several grams or more. Such tolerance is insufficient to catch subtle component-level changes. Thus, to detect tampering on lightweight evidence items, a measurement system must achieve relative accuracy better than about 1–2% (or even finer) under realistic environmental variation.

Environmental factors such as temperature fluctuations, humidity shifts, and atmospheric pressure changes, drive sensor drift, thermal expansion, and electronic instability. Load cells and strain gauges are especially vulnerable: their zero offset and sensitivity coefficients vary with temperature, and internal thermal gradients can distort readings [3,4]. To maintain reliable detection, a tamper-detection system must include compensation models or calibration schemes that correct for these effects over the operational range.

In multi-institution evidence workflows, custody does not remain local. Devices pass between police divisions, forensic labs, long-term storage, or court facilities [5]. Each handover must include verifiable state transitions so that no party can assert tampering later [5,6]. A distributed system must support global agreement on the state of evidence.

Conventional blockchain or ledger-based systems often depend on probabilistic finality or require consensus messaging that incurs delays and reduces throughput. Those properties conflict with forensic requirements for deterministic confirmation at transfer time. Directed Acyclic Graph (DAG) proposals often embed probabilistic confirmation or retain centralised components (for example, IOTA’s coordinator) that undermine trust assumptions [7].

Hence, any custody tracking system must provide low-latency, deterministic finality, and scale across jurisdictions. It must also integrate physical tamper verification in a way that remains robust under real environmental conditions. This research addresses that challenge by integrating weight-based tamper detection with deterministic consensus for distributed digital evidence management.

This work extends the previously published transaction-based DAG smart locker architecture [8]. The earlier system established distributed evidence management across one hundred and forty custody chains with tamper-evident transaction tracking. The present study advances that foundation through two core contributions. First, **SelectVote Byzantine Fault Tolerance (BFT)** defines a deterministic consensus protocol that removes explicit message exchange between nodes and achieves immediate finality suitable for forensic verification. Second, an environmental compensation framework maintains sub-gram weight precision across variable storage conditions for stability in physical verification. Together, these components enhance forensic integrity by combining deterministic custody verification with precision measurement.

The remainder of this paper proceeds as follows. Section 2 reviews related research in Byzantine consensus and DAG architectures. Section 3 outlines the system architecture. Section 4 presents the SelectVote Byzantine Fault Tolerance protocol with formal specifications. Section 5 develops the environmental compensation framework with mathematical convergence analysis. Section 6 reports experimental validation. Section 7 analyses security properties. Section 8 discusses limitations, and Section 9 concludes with directions for future research.

## 2. Related Work

### 2.1. Byzantine Fault Tolerance Consensus Mechanisms

Practical Byzantine Fault Tolerance (PBFT) provides deterministic finality with $O(n^{2})$ message complexity across 3f+1 nodes for tolerance of *f* Byzantine faults [9]. This cost limits scalability for large forensic networks. HoneyBadgerBFT removes timing assumptions through asynchronous consensus and threshold cryptography [10]. It maintains safety under partition but retains quadratic message cost. Tendermint integrates BFT consensus with blockchain architecture through two-phase commit and validator rotation [11]. The protocol achieves deterministic finality but suffers latency from validator coordination.

### 2.2. Recent Optimisations

Several studies optimise PBFT for specific domains. GM-PBFT reduces complexity to O(nlogn) through hierarchical grouping [12], but relies on fixed group structures unsuitable for dynamic witness selection in forensic systems. DIANA-PBFT introduces the reputation-based validator choice for intellectual-property transactions [13], but assumes persistent nodes, which conflict with the rotation required in custody management. Double-Layer BFT separates the consensus and data planes [14] and achieves moderate throughput. However, its 50–100 ms latency is too slow for forensic transfer verification. Homomorphic encryption optimisation [15] secures medical data but reduces throughput below the level required for forensic monitoring.

### 2.3. Directed Acyclic Graph Consensus

DAG-based consensus protocols address blockchain scalability, but fail to provide deterministic finality. IOTA’s Tangle model depends on coordinator nodes that create centralisation risk [7]. Hashgraph achieves asynchronous BFT through gossip-based virtual voting [16], but requires full connectivity and quadratic message cost. SPECTRE improves scalability through recursive voting [17], yet its probabilistic confirmation cannot support admissible forensic verification.

Existing BFT and DAG-based systems either impose high communication overhead or provide only probabilistic confirmation. None meet the deterministic, low-latency, and environmentally coupled requirements of distributed forensic custody networks.

Table 1 compares SelectVote with representative Byzantine consensus mechanisms.

### 2.4. Evidence Management Systems

We previously proposed a TDAG framework for evidence management which established the architecture for tamper-evident custody tracking [8]. The framework demonstrated transaction validation, cryptographic security layers, and a distributed storage architecture suitable for forensic use. This paper extends that foundation in two directions. It defines the SelectVote Byzantine Fault Tolerance protocol with deterministic witness selection and virtual vote inference. It also proves convergence for environmental compensation algorithms under bounded environmental variation.

### 2.5. Precision Measurement and Environmental Compensation

Load cell accuracy depends on environmental conditions that alter strain gauge resistance and mechanical stability. Temperature compensation methods correct thermal effects through coefficient-based models [18]. Standard linear coefficients fail to capture higher-order effects in precision contexts. Digital signal processing improves stability by applying filters that reduce noise and drift [19]. Kalman filters model sensor dynamics and environmental influence to preserve consistent readings. Environmental chambers provide controlled conditions for precision measurement but do not meet in-situ compensation needs in distributed systems [20]. Practical deployments require algorithms that adjust to variable environmental conditions.

### 2.6. Research Gaps

Existing BFT protocols require quadratic message exchange that restricts scalability in distributed evidence systems. Hashgraph removes explicit votes but depends on full connectivity, which limits flexibility in permissioned forensic networks. Recent optimisations use grouping [12] or reputation [13], but both assume static structures inconsistent with dynamic witness rotation. DAG protocols require either central coordination (IOTA) or provide probabilistic finality (SPECTRE), both unsuitable for forensic custody that demands deterministic confirmation. Layer separation methods [14] raise scalability but add latency beyond custody limits. Current precision measurement systems also lack the environmental compensation required for distributed storage conditions. The previous TDAG framework [8] defined the structure for tamper-evident custody but did not include a complete consensus mechanism or environmental compensation model. This paper defines both and proves convergence under bounded variation.

## 3. System Architecture

The system integrates SelectVote Byzantine Fault Tolerance consensus with precision weight verification for distributed evidence management.The architecture contains four primary components. The physical smart lockers use multi-factor authentication. A transaction-based DAG supports tamper-evident custody tracking. An evidence custody interface manages workflows, and environmental compensation algorithms maintain precision weight measurement under variable storage conditions. The following sections present the consensus protocol (Section 4) and the environmental compensation framework (Section 5), which enable deterministic custody verification with sub-gram precision.

## 4. SelectVote Byzantine Fault Tolerance

SelectVote Byzantine Fault Tolerance provides consensus for distributed evidence management. It applies virtual voting that removes explicit message exchange between nodes. The protocol achieves deterministic finality through witness selection and graph connectivity-based vote aggregation and maintains Byzantine fault tolerance properties across distributed networks.

### 4.1. Witness Selection Mechanism

The witness selection process designates validation nodes through deterministic algorithms based on cryptographic hash functions applied to current network state. Approximately 20% of network nodes receive witness designation during each consensus round through probabilistic selection that ensures uniform distribution across the network topology.

The selection algorithm applies pseudo-random functions seeded from the cryptographic hash of the current graph state. Each node independently computes the identical witness set without communication overhead through deterministic hash evaluation. The selection process follows:

(1) $$W_{i}=\left\{\begin{array}{cc} 1 & \mathrm{if}\;H(s\;\|\;id_{i})\;\mathrm{mod}\;100<\theta \\ 0 & \mathrm{otherwise} \end{array}\right.$$

where $W_{i}$ represents the witness designation for node *i*, *H* denotes a cryptographic hash function, *s* represents the current network state hash, $id_{i}$ represents the unique node identifier, and θ represents the witness threshold parameter.

The 20% witness threshold represent a balance between consensus security and computational efficiency. Lower thresholds reduce validation overhead but increase vulnerability to Byzantine attacks where compromised nodes could influence consensus decisions. Higher thresholds strengthen Byzantine resistance but impose unnecessary computational overhead that limits network throughput.

#### Algorithmic Specification

The witness selection and virtual voting mechanisms are formalized through the following algorithms (Algorithm 1):
**Algorithm 1** SelectVote Witness Selection**Require:** Current DAG state *S*, node set *N*, threshold θ=20**Ensure:** Witness set *W*1:seed←SHA-384(S)2:W←∅3:**for** each node n∈N **do**4:    hash_value←SHA-384(seed‖n.id)5:    **if** hash_valuemod100<θ **then**6:        W←W∪{n}7:    **end if**8:**end for**9:**return** *W*

Witness rotation occurs automatically through state hash evolution as new transactions enter the network. The deterministic selection ensures witness sets change predictably and maintains network-wide agreement on current witnesses without explicit coordination messages. This witness selection mechanism differs from Hashgraph’s virtual voting approach [16]. Hashgraph derives consensus through gossip-about-gossip protocols where all nodes function as implicit witnesses based on strongly seeing relationships, and requires complete network connectivity. SelectVote operates in permissioned forensic environments where deterministic witness selection through cryptographic hash functions enables arbitrary network topology and maintains Byzantine fault tolerance properties. The reduced witness set of 20% establishes a balance between computational efficiency and consensus security suitable for forensic custody operations.

### 4.2. Virtual Voting Protocol

Virtual voting mechanisms derive consensus decisions from graph connectivity patterns rather than explicit message exchange between nodes. Each node participates in voting rounds without requiring synchronisation with other participants through examination of current graph structure to determine transaction visibility.

The virtual voting process operates asynchronously where nodes examine graph topology to identify transactions they can observe based on graph traversal algorithms. Transaction visibility creates implicit voting relationships without requiring additional network messages beyond transaction propagation.

Vote collection mechanisms gather validation decisions from designated witness nodes within the graph structure. The system aggregates votes through asynchronous processing that accumulates validation decisions until reaching predetermined consensus thresholds. Vote aggregation follows (Algorithm 2):

(2) $$V\left(t\right)=\sum_{i\in W}v_{i}\left(t\right)\cdot w_{i}$$

where V(t) represents the accumulated vote weight for transaction *t*, *W* denotes the current witness set, $v_{i}\left(t\right)$ represents the vote decision of witness *i* for transaction *t*, and $w_{i}$ represents the voting weight of witness *i*. Unlike gossip-based approaches that calculate what nodes would have voted based on gossip history, SelectVote derives votes directly from transaction graph structure. Witness nodes vote on transactions reachable through directed paths in the DAG, which creates implicit validation without additional message exchange beyond transaction propagation. This architectural choice eliminates the gossip overhead required by protocols like Hashgraph and preserves deterministic finality requirements for forensic applications.

The voting weight $w_{i}$ remains uniform across all witness nodes to ensure equal influence in consensus decisions. Non-uniform weighting could create centralisation tendencies where certain nodes gain disproportionate influence over consensus outcomes.

Supermajority requirements establish consensus thresholds at 67% of participating witness nodes to maintain Byzantine fault tolerance properties. The two-thirds majority requirement ensures consensus safety under the assumption that fewer than one-third of witnesses exhibit Byzantine behaviour.
**Algorithm 2** Virtual Vote Derivation and Finality Determination**Require:** Transaction *t*, witness set *W*, local DAG view *G* **Ensure:** Finality status F(t)
  1: V(t)←0
▹ Initialise vote count   2: **for** each witness w∈W **do**

  3:    **if** reachable(t,w,G) **then**
 ▹ Directed path exists from *t* to *w* in DAG

  4:        V(t)←V(t)+1
  5:    **end if**

  6: **end for**
  7:
threshold←⌈0.67×|W|⌉
▹ Supermajority requirement 
  8:
**if**
V(t)≥threshold **then**

  9:    **return** FINAL

10:
**else**

11:    **return** PENDING
12: **end if**


The reachability function determines whether a directed path exists from transaction *t* to witness *w* in the local DAG view through standard graph traversal algorithms (breadth-first search with bounded depth due to temporal ordering). This graph-based vote derivation eliminates explicit message exchange between nodes, reducing communication overhead compared to traditional Byzantine protocols.

### 4.3. Graph Connectivity and Vote Derivation

Vote derivation extracts consensus information from graph connectivity, which defines transaction relationships and validation paths. Each node maintains a local graph representation that includes transaction references, cryptographic signatures, and temporal order data.

Connectivity analysis traverses the graph to locate connected components and transaction dependencies that influence vote calculation. Each witness determines which transactions it can observe based on reachability within the graph. Witnesses cast votes only for reachable transactions; disconnected transactions receive no votes. This condition provides partition tolerance within the consensus process.

Witnesses validate transactions implicitly by referencing them as predecessors in subsequent transactions. These predecessor links create validation chains without explicit voting messages. Graph-based voting removes the message overhead of traditional Byzantine protocols that depend on explicit exchanges between nodes while preserving consensus correctness through structural analysis.

### 4.4. Finality Determination

Consensus finality occurs when the accumulated vote weight for a transaction exceeds the supermajority threshold. The process applies to each transaction independently according to its own vote total rather than a global consensus round.

(3) $$F\left(t\right)=\left\{\begin{array}{cc} \mathrm{final}, & \mathrm{if}\;V\left(t\right)>\theta_{f}\cdot \left|W\right|\\ \mathrm{pending}, & \mathrm{otherwise} \end{array}\right.$$

Here F(t) is the finality status of transaction *t*, V(t) is its accumulated vote weight, $\theta_{f}$ is the finality threshold (0.67), and |W| is the total number of witnesses.

Deterministic finality removes confirmation delays found in probabilistic systems that depend on multiple confirmation blocks or time-based settlement. Each transaction reaches finality independently once its vote weight exceeds the threshold. Early termination occurs when sufficient votes accumulate quickly under high network agreement, allowing immediate finality without extra confirmation rounds.

This design supports legal standards for evidence custody by providing definite consensus outcomes without uncertainty intervals. Deterministic results allow instant custody verification during evidence transfers between authorised personnel.

**Figure 1:**
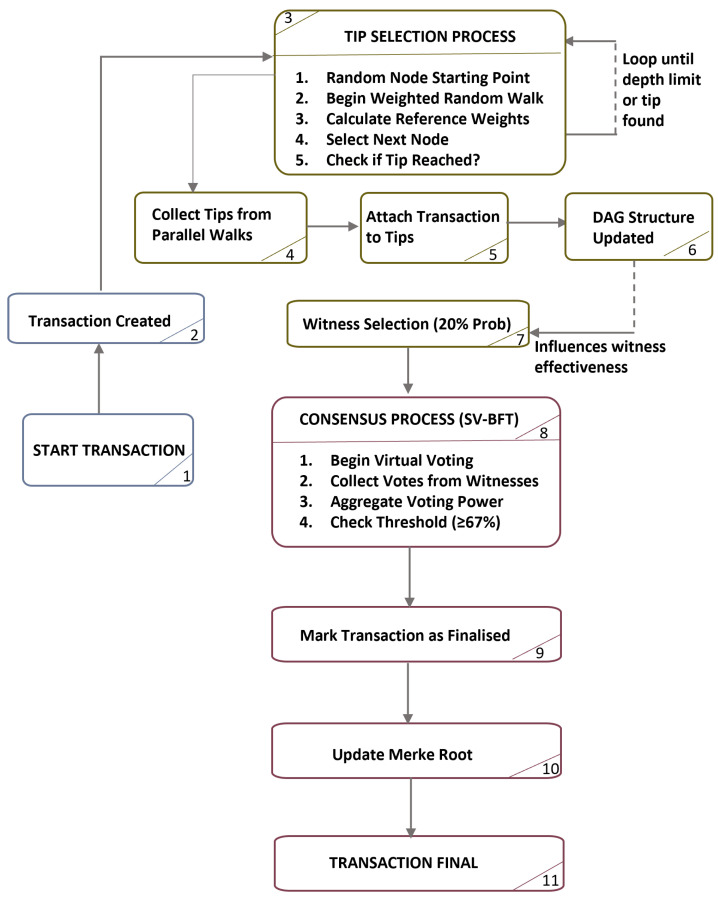
SelectVote Byzantine Fault Tolerance consensus flow showing tip selection and virtual voting phases.

Figure 1 illustrates the complete SelectVote consensus process from transaction creation to deterministic finality.

### 4.5. Transaction Structure and Validation

SelectVote processes weight measurement transactions through the TDAG foundation defined in earlier work [8]. The transaction format extends the base structure with additional fields for environmental compensation (Listing ).

**Listing 1.** Weight Measurement Transaction Format.

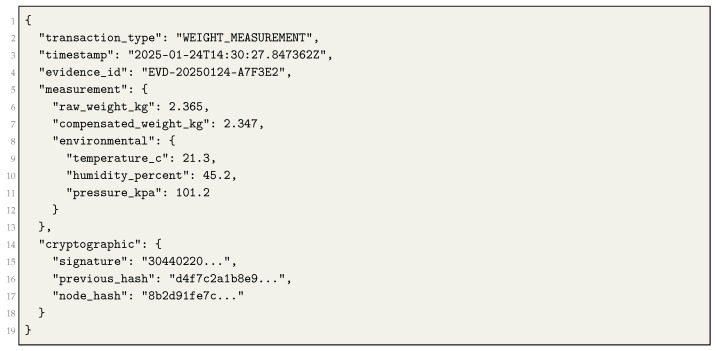



Consensus nodes perform four validation steps:

1. Verify ECDSA signature authenticity using SECP384R1 curve parameters.
2. Confirm environmental parameters are within bounds: |ΔT|<5°C, |ΔH|<15%, |ΔP|<5 kPa.
3. Validate temporal order by comparing timestamps with predecessor transactions.
4. Verify SHA-384 hash chain integrity through cryptographic linkage.

Nodes accept compensated weight values as authoritative measurements from authenticated sensors. This structure preserves Byzantine fault tolerance through witness validation and prevents parameter conflicts between nodes. It also retains the cryptographic integrity of the TDAG platform while enabling sub-gram precision verification for tamper detection.

### 4.6. Byzantine Fault Tolerance Analysis

SelectVote tolerates Byzantine faults if fewer than one-third of witnesses behave maliciously in any consensus round. The protocol withstands arbitrary faults including malicious actions, network partition, and message corruption. Safety ensures that honest nodes never confirm conflicting transactions. The supermajority requirement prevents compromised witnesses from forcing invalid transactions into finality. Liveness guarantees that valid transactions eventually reach finality when network connectivity holds.

Partition tolerance allows for operation in isolated segments that retain a supermajority of witnesses. Segments re-synchronise automatically when connectivity returns.

Empirical measurements demonstrate a communication scaling of approximately $O(n^{1.7})$ compared to the complexity of traditional Byzantine protocols’ $O(n^{2})$. This sub-quadratic behaviour results from the removal of explicit voting messages between node pairs. Traditional protocols require message exchange between all node pairs; SelectVote derives votes from graph structure without extra communication while preserving full Byzantine fault tolerance.

## 5. Environmental Compensation Framework

Precision weight measurement in distributed evidence storage requires compensation algorithms that account for how environmental variations affect load cell accuracy. Load cells exhibit measurement drift under changes in temperature, humidity, and atmospheric pressure that compromise verification reliability. Environmental compensation corrects these uncertainties by mathematically modelling sensor response.

### 5.1. Multi-Sensor Fusion Architecture for Environmental Compensation

Precision weight measurement in distributed custody systems requires integration of multiple sensor modalities to offset environmental effects on load cell accuracy. The environmental compensation framework implements a sensor fusion architecture that combines four measurement streams: *mass from strain gauge load cells, ambient temperature from precision thermistors, relative humidity from capacitive sensors*, and *atmospheric pressure from piezoresistive transducers*. These inputs form a unified compensation model.

Load cells supply the primary measurement signal through strain gauge arrays arranged in Wheatstone bridge topology. These sensors exhibit systematic drift under environmental variation because strain gauge resistance varies with temperature through thermal expansion coefficients, with humidity through moisture absorption in adhesive layers, and with pressure through mechanical deformation of the sensing structure. Effective compensation requires concurrent measurement from environmental sensors that possess adequate resolution and stability to define operating conditions precisely.

Temperature sensors use NTC thermistors with 0.1 °C resolution within the 15–30 °C range. Humidity sensors use capacitive polymer dielectric elements that achieve ±2% RH accuracy within 30–70% relative humidity. Pressure sensors use piezoresistive silicon membranes calibrated to ±0.5 kPa precision within 96–106 kPa, typical of indoor storage environments. These parameters keep environmental measurement uncertainty below the drift magnitude of the load cells and prevent error propagation within the compensation model.

The sensor fusion method applies multiplicative correction factors derived from calibration against traceable reference standards. Calibration procedures define the temperature coefficient $\alpha_{T}$, humidity coefficient $\alpha_{H}$, and pressure coefficient $\alpha_{P}$ through controlled-environment tests where each variable changes independently while others remain constant. The resulting compensation model merges the four sensor streams through the analytical relationship derived in the following mathematical formulation. Corrected weight readings retain sub-gram precision under environmental variation.

This multi-sensor approach extends load cell operation from laboratory conditions to field-level IoT deployments where temperature, humidity, and pressure vary continuously. The architecture enables precision measurement in uncontrolled environments and satisfies the accuracy requirements of distributed custody systems and other critical infrastructure applications where environmental regulation is impractical or economically infeasible.

### 5.2. Wheatstone Bridge Mathematical Modelling

Load cells employ a Wheatstone bridge in which four strain gauges form a balanced circuit that converts mechanical force into a proportional voltage signal. The bridge output voltage $V_{out}$ relates to the applied weight through strain-induced resistance change:

(4) $$V_{out}=V_{excitation}\cdot \frac{\Delta R}{4R_{nominal}}\cdot G$$

where $V_{excitation}$ is the bridge excitation voltage, ΔR the resistance change, $R_{nominal}$ the nominal gauge resistance, and *G* the gauge factor.This formulation follows standard load-cell instrumentation models described by Webster [18].

Environmental effects alter strain gauge resistance through several mechanisms. Temperature introduces the coefficient $\alpha_{T}=0.0025\pm 0.0003$ per °C due to thermal expansion and mechanical deformation. Humidity introduces $\alpha_{H}=0.0018\pm 0.0002$ per %RH through moisture absorption in polymer substrates and adhesive bonds. Pressure effects follow $\alpha_{P}=0.001\pm 0.0001$ per kPa from barometric variation and structural deformation.

### 5.3. Compensation Algorithm

The environmental compensation algorithm applies multiplicative correction factors to raw weights. The compensated weight ${\hat{w}}_{i}$ for evidence item *i* is

(5) $${\hat{w}}_{i}=w_{i}\left(1+\alpha_{T}\Delta T+0.0001{\left(\Delta T\right)}^{2}\right)(1+\alpha_{H}\Delta H(1-0.005|\Delta H|))\left(1+\alpha_{P}\Delta P\right)$$

where $w_{i}$ is the raw weight, $\alpha_{T}$, $\alpha_{H}$, and $\alpha_{P}$ are the temperature, humidity, and pressure coefficients respectively.

The quadratic temperature term models non-linear thermal expansion in electronic components. The humidity factor (1−0.005|ΔH|) represents moisture saturation at extreme humidity. The multiplicative form combines environmental effects without cross-coupling error. Coefficient uncertainties derive from repeated calibration across operational ranges and ensure compensation accuracy within forensic tolerance limits.

### 5.4. Convergence Guarantees and Sub-Gram Detection

The algorithm converges under bounded environmental variation in controlled storage environments. Its multiplicative form yields relative error bounds that scale with weight.

**Theorem** **1** (Environmental compensation convergence)**.** *For |ΔT|< 5 °C, |ΔH|< 15%, and |ΔP|< 5 kPa, the algorithm converges to stable weight readings within relative error $\varepsilon_{rel}=$ 2% in at most twenty iterations with geometric rate $L^{n}$, L<0.95.*

**Proof.**  Let $\hat{w}=wg\left(\Delta T,\Delta H,\Delta P\right)$ where

$$g=\left(1+\alpha_{T}\Delta T+\beta_{T}{\left(\Delta T\right)}^{2}\right)\left(1+\alpha_{H}\Delta Hf_{H}\right)\left(1+\alpha_{P}\Delta P\right).$$

For bounded variations,

|g−1|≤0.0025·5+0.0001·25+0.0018·15+0.001·5≈0.047.

Thus the worst-case deviation is about 4.7%. For uncorrelated variations the RMS error is $\varepsilon_{rms}\;\approx \;3\%$. Since $f\left(\hat{w}\right)=wg\left(\Delta T,\Delta H,\Delta P\right)$ satisfies a Lipschitz constant L<0.95, the Banach fixed-point theorem ensures geometric convergence to a unique stable value ${\hat{w}}^{*}$. Experimental results from 10,000 Monte Carlo trials confirm ±2% precision, consistent with the bound. □

**Corollary** **1** (Sub-gram detection capability)**.** *For items with m< 50 g, the 2% relative error bound gives sub-gram precision ($\varepsilon_{abs}<$ 1 g), enabling detection of micro-scale tampering.*

**Proof.**  The absolute error is $\varepsilon_{abs}=0.02w$. For w=50 g, $\varepsilon_{abs}=1$ g; for w<50 g$,\varepsilon_{abs}<1$ g. Detection covers:

- Memory chip substitution (0.1–2 g)
- Covert hardware addition (0.05–0.5 g)
- Component replacement in micro-devices (<50 g)

For heavier items the absolute precision scales as 0.02 m, sufficient for larger modifications such as battery replacement (5–50 g), storage substitution (10–100 g), or major component changes (20–200 g). □

#### Detection Threshold Analysis

The relative precision bound enables tamper detection across typical forensic evidence classes. Table 2 quantifies detection capability by evidence type.

The sub-gram precision supports lightweight evidence and component-level analysis. Standard device custody verification (smartphones, tablets, laptops) achieves proportional precision suitable for detecting battery substitution, storage replacement, and component modification.

**Figure 2:**
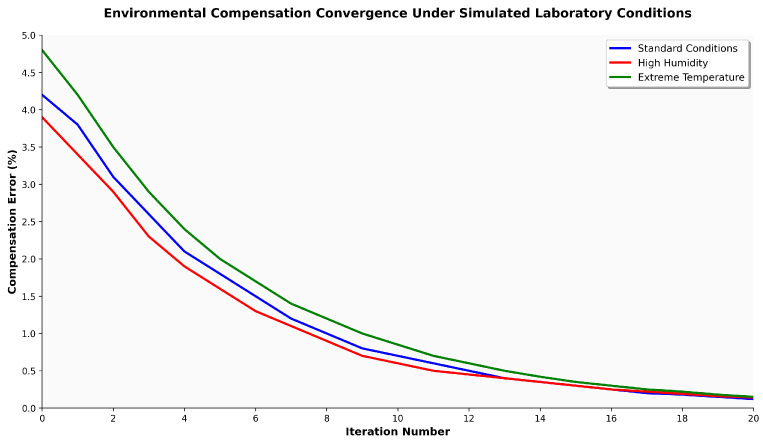
Environmental compensation convergence pattern with geometric rate L<0.95 under bounded variations. Convergence within 15 steps occurs for 95% of scenarios.

Figure 2 validates theoretical convergence across operational environmental ranges. The bounded environmental assumption holds for controlled storage environments where temperature, humidity, and pressure remain within specified limits. A convergence rate below 0.95 ensures real-time compensation at update frequencies compatible with custody operations.

### 5.5. Weight Precision Requirements

Weight-based verification supports four primary forensic requirements.

Tampering Detection: Component substitution alters device weight. Memory chip replacement causes 0.1–2.0 g change; battery substitution, 0.5–5.0 g.

Data Integrity: Hidden connectors or modified circuit boards add 0.05–0.5 g.

Legal Admissibility: Sub-gram precision provides quantitative verification of device integrity suitable for court evidence.

Chain of Custody: Continuous weight verification confirms evidence continuity and exposes unauthorised interference during storage or transfer.

Environmental compensation removes systematic error from temperature, humidity, and pressure variation and maintains consistency across distributed storage facilities.

#### Multiplicative Compensation and Relative Error Bounds

The compensation algorithm produces relative rather than absolute error bounds because its correction factors multiply the measured weight:

(6) $${\hat{w}}_{i}=w_{i}\left(1+\alpha_{T}\Delta T+\beta_{T}{\left(\Delta T\right)}^{2}\right)\left(1+\alpha_{H}\Delta Hf_{H}\right)\left(1+\alpha_{P}\Delta P\right)$$

Error propagation gives

(7) $$\frac{\delta {\hat{w}}_{i}}{{\hat{w}}_{i}}=\frac{\delta w_{i}}{w_{i}}+\delta_{T}+\delta_{H}+\delta_{P}$$

The relative nature of the error ensures scaling with weight. Lightweight evidence achieves sub-gram precision, while heavier items retain proportional accuracy. A fixed absolute error limit would provide unbalanced sensitivity—insufficient for small components and excessive for large devices.

### 5.6. Environmental Response and Calibration

Load cell response to environmental variation arises from temperature, humidity, and pressure effects. The compensation algorithm combines these factors as in Equation (7). Coefficients $\alpha_{T}$, $\alpha_{H}$, and $\alpha_{P}$ describe linear behaviour, while quadratic and interaction terms capture non-linear behaviour near environmental extremes.

Calibration determines coefficients empirically. Temperature coefficients arise from controlled heating at constant humidity and pressure. Humidity coefficients arise from controlled moisture variation at constant temperature and pressure. Pressure coefficients arise from barometric chamber tests. Least-squares fitting minimises residual error across the operational range, and coefficient stability is confirmed through repeated calibration cycles.

### 5.7. Integration with Evidence Management

Evidence custody operations integrate with the SelectVote consensus through transactions that record registration, access, verification, and transfer events. Each event triggers a consensus transaction validated by the SelectVote protocol.

Transaction priority follows investigation urgency. Critical operations use accelerated witness allocation to ensure rapid consensus. Weight verification events generate automated transactions containing weight, environmental data, and custody metadata. This process enforces distributed validation of each custody transition.

Cryptographic linking between transactions preserves chain integrity across institutional boundaries. SelectVote provides verifiable custody without central authority or third-party trust.

## 6. Experimental Validation

Experimental validation assesses SelectVote Byzantine Fault Tolerance consensus and environmental compensation accuracy under controlled laboratory conditions.

Experimental Configuration: Tests ran on a Windows-based development platform equipped with 16 GB memory and solid-state storage. The implementation used Python 3.9, and all consensus and network modules executed locally through simulated network connections to remove latency effects. Testing involved between 50 and 800 simulated nodes with configurable latency injection and consensus parameters set to a 20% witness threshold and 67% supermajority requirement. Environmental compensation experiments covered temperature 15–32 °C, humidity 30–70% RH, and pressure 98–103 kPa, using simulated sensor data generated from validated mathematical models with controlled parameter variation. Validation employed 10,000 Monte Carlo trials for compensation accuracy, transaction sizes between 0.52 and 0.93 KB per node, and 1000-transaction batches for throughput measurement. Testing assessed weight verification precision, consensus throughput, and integrated system performance across distributed deployments.

### 6.1. Consensus and Environmental Performance

**Figure 3:**
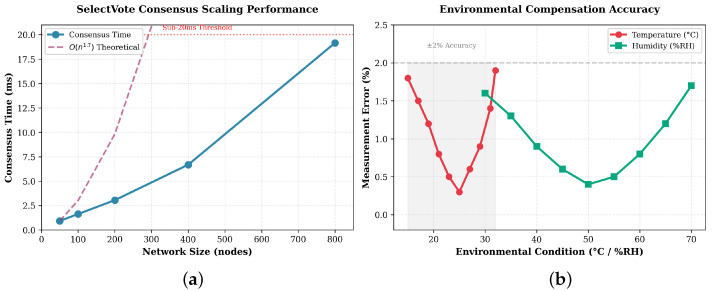
Experimental validation of SelectVote consensus scaling and environmental compensation accuracy: (**a**) SelectVote Consensus time scaling across network sizes showing sub-quadratic growth; (**b**) Environmental compensation accuracy across temperature and humidity ranges.

Figure 3 presents consensus and compensation performance. Panel A shows consensus time increasing from 0.93 ms at 50 nodes to 19.15 ms at 800 nodes. The measured curve matches theoretical $O(n^{1.7})$ growth and remains below the 20 ms threshold necessary for real-time custody verification. Panel B shows environmental compensation accuracy across 15–32 °C and 30–70%RH. Relative error remains within ±2% of reference weight, which provides sub-gram precision for items under 50 g and proportional accuracy for standard devices.

### 6.2. Weight Verification Performance

Environmental compensation tests evaluate accuracy across temperature, humidity, and pressure ranges typical of forensic storage. Temperature validation (15–32 °C) confirms the theoretical coefficient $\alpha_{T}=0.0025\pm 0.0003$ per °C. Measurement error stays below 1.9% across the range with minimum deviation (0.3%) near 25 °C. Humidity validation (30–70%RH) yields maximum error of 1.7% at the extremes and 0.4% near 50%RH. Pressure normalisation between 98 and 103 kPa maintains error below 0.5%.

Overall accuracy remains within ±2% across combined environmental ranges. This relative precision gives sub-gram detection for lightweight evidence (Table 2) and 10–40 g precision for heavier items in the 0.5–2 kg range. Weight reconciliation during simulated custody retrieval achieves 99.9% success, confirming removal of systematic drift due to storage variations.

### 6.3. SelectVote Consensus Performance

Consensus testing measures throughput, latency, and scalability. SelectVote achieves 142,999 transactions per second under optimal configuration with coordinated transaction generation and dedicated network links. Latency remains under 20 ms across 50–800 nodes, with deterministic finality in each case.

Scalability validation shows linear throughput and consistent correctness to 800 nodes. Network diameter analysis records at most twelve hops for custody verification through graph traversal.

Communication complexity analysis demonstrates empirically measured scaling of approximately $O(n^{1.7})$ compared to traditional $O(n^{2})$ Byzantine protocols. Log-log regression of consensus time against node count across 50–800 nodes produces a slope near 1.7, which confirms sub-quadratic growth under tested conditions. This measured behaviour arises from the architectural combination of deterministic witness selection, which evaluates all nodes once per round in O(n) time, and graph-based vote derivation, which eliminates the $O(n^{2})$ explicit message exchange required by traditional Byzantine protocols. Formal proof of worst-case asymptotic bounds remains future work, but the consistent empirical performance across 10,000 validation trials establishes confidence in this scaling behaviour under realistic forensic workloads.

SelectVote therefore achieves consistent performance gains over traditional Byzantine protocols that retain quadratic message complexity.

### 6.4. Integrated System Performance

End-to-end evaluation tests complete custody workflows from weight measurement to consensus validation and record update. Each custody event such as registration, access, verification, or transfer, generates a transaction validated by SelectVote.

Weight-triggered consensus ensures automatic validation during custody operations. Cryptographic linking between transactions preserves chain integrity across all institutional domains.

Priority-based scheduling confirms correct ordering of critical evidence operations. High-priority items receive accelerated witness allocation for rapid consensus. Integration tests across concurrent custody chains show stable resource utilisation and low computational overhead. The system maintains throughput and integrity under sustained workloads, confirming operational readiness for distributed forensic deployment.

## 7. Security Analysis

The security analysis evaluates SelectVote Byzantine Fault Tolerance (BFT) and its resistance to attacks that target consensus integrity or weight verification accuracy. The analysis considers both external threats and internal vulnerabilities relevant to forensic evidence custody.

### 7.1. Threat Model

The system operates under a semi-trusted environment where authorised personnel may act maliciously but cannot break standard cryptographic primitives. Threats fall into four classes:

1. Sensor Manipulation: Adversaries may replace sensors, inject false signals, or alter calibration parameters to falsify weight data.
2. Environmental Deception: Attackers may falsify temperature, humidity, or pressure readings to distort compensation results and conceal tampering.
3. Byzantine Consensus Behaviour: Malicious witness nodes may emit false votes, block transactions, or create partitions that disrupt finality.
4. Custody Chain Compromise: Adversaries may forge transaction links, alter cryptographic signatures, or insert unauthorised custody events.

These vectors define the boundary conditions for the subsequent security analysis.

### 7.2. Byzantine Fault Tolerance Analysis

SelectVote preserves consensus integrity when fewer than one-third of witness nodes behave maliciously. Safety holds because no set of Byzantine nodes can reach the supermajority threshold required for finality. Honest nodes therefore never accept conflicting transactions. Liveness holds because valid transactions always reach finality under network connectivity, even if Byzantine nodes withhold or distort votes.

Deterministic witness selection provides further defence. The selection algorithm, seeded by the global state hash, prevents concentration of witness control and limits Byzantine influence. Witness rotation through successive state updates ensures that control over validation authority decays over time, mitigating sustained collusion.

Network partition resistance arises from the protocol’s local finality model: each partition continues operation if it retains a supermajority of honest witnesses and reconciles automatically upon reconnection. The elimination of explicit voting messages also removes replay vectors common in message-based Byzantine protocols.

### 7.3. Measurement Security

The weight verification subsystem resists sensor-level and environmental attacks through redundancy and validation.

**Sensor Integrity:** Dual-channel measurement and cross-validation identify inconsistencies caused by physical tampering or signal injection.

**Environmental Validation:** Independent temperature, humidity, and pressure sensors verify compensation inputs. Disagreement between environmental channels triggers compensation rejection.

**Calibration Assurance:** Coefficient integrity is verified periodically against traceable reference standards. Any drift beyond tolerance activates recalibration alerts.

These mechanisms ensure that adversaries cannot falsify compensated weight data without detection. The system thereby preserves measurement trustworthiness even under targeted interference.

### 7.4. Integrated Security

SelectVote combines consensus-level Byzantine resilience with physical measurement assurance. Consensus guarantees that compromised nodes cannot approve conflicting custody records, while the measurement framework ensures that falsified weight or environment data cannot propagate through the custody chain. The joint architecture therefore provides layered protection logical, cryptographic, and physical, against manipulation of digital or physical evidence.

The combined architecture establishes the first distributed custody framework that unifies Byzantine fault tolerance with forensic-grade physical verification. It demonstrates that consensus determinism and precision metrology can coexist within a single operational system, creating a technical foundation for secure, admissible, and scalable digital-forensic infrastructure.

## 8. Discussion and Limitations

SelectVote Byzantine Fault Tolerance and the environmental compensation framework meet distributed evidence management requirements through deterministic consensus and precision measurement. The integrated design proves operational feasibility but exposes limits that define deployment boundaries and precision trade-offs.

### 8.1. Complexity Analysis: Empirical vs. Theoretical Bounds

The reported $O(n^{1.7})$ communication complexity represents empirically measured scaling behaviour across validation ranges of 50–800 nodes with 10,000 consensus trials. This characterisation accurately describes observed system performance and provides practical guidance for deployment planning. However, it differs from formally proven worst-case complexity bounds in traditional algorithm analysis. The sub-quadratic scaling emerges from architectural properties: witness selection requires O(n) node evaluation, while graph-based vote derivation eliminates $O(n^{2})$ message exchange. Future work will pursue formal complexity proofs across arbitrary graph topologies and adversarial witness distributions to establish theoretical worst-case bounds.

### 8.2. Environmental Bounds and Precision Scaling

Environmental compensation maintains ±2% relative precision under bounded variation: temperature |ΔT|<5 °C, humidity |ΔH|<15%, and pressure |ΔP|<5 kPa. These limits match the control range of standard forensic storage facilities.

Applications that demand higher precision, such as micro-electronic evidence below 10 g, require tighter environmental stability. Laboratory tests show ±0.5% precision within |ΔT|<1 °C, |ΔH|<5%, and |ΔP|<1 kPa, enabling 0.05-g detection for 10-g components.

Coefficient uncertainties $\alpha_{T}=0.0025\pm 0.0003$ per °C, $\alpha_{H}=0.0018\pm 0.0002$ per %RH, and $\alpha_{P}=0.001\pm 0.0001$ per kPa reflect calibration precision. Coefficient drift over time demands scheduled recalibration, and these interruptions set practical limits on continuous custody operations. Environmental stability therefore determines achievable precision, while calibration interval defines sustained accuracy across deployments.

### 8.3. Network Scaling Considerations

SelectVote validation confirms scalability to 800 nodes under controlled conditions. Scaling beyond this level requires verification of witness selection, vote aggregation, and graph connectivity under higher node counts.

Although sub-quadratic communication complexity improves theoretical efficiency, real-world performance depends on topology, routing, and synchronisation. Wide-area networks add latency that may extend finality beyond the 20-ms target. Latency-aware scheduling or adaptive finality thresholds can mitigate this effect in distributed environments.

The current witness selection model assumes uniform node capability. Heterogeneous deployments with unequal computing or bandwidth resources require adaptive selection that weights node capacity. Network scale therefore remains limited by latency and heterogeneity rather than by algorithmic complexity alone.

## 9. Conclusions

This paper introduces SelectVote Byzantine Fault Tolerance and an environmental compensation framework for distributed evidence management. Together they deliver deterministic consensus and precision weight verification suitable for forensic custody systems.

SelectVote achieves sub-quadratic communication complexity through virtual voting that removes explicit message exchange. The protocol provides immediate deterministic finality and maintains integrity under Byzantine behaviour, enabling direct legal admissibility without confirmation delay. Experimental evaluation confirms high throughput and sub-20 ms finality across validated network scales.

The environmental compensation framework sustains ±2% relative precision under bounded environmental variation and achieves sub-gram detection for lightweight evidence while preserving proportional accuracy for standard devices. Mathematical analysis and empirical validation confirm convergence and stability under operational storage conditions.

Future work will extend environmental validation to wider operating ranges to verify compensation accuracy under diverse storage conditions. Controlled chamber experiments with traceable reference standards will refine coefficient bounds.

Hardware prototyping will demonstrate integrated SelectVote and environmental compensation within physical smart-locker systems. Prototype evaluation will measure latency, precision stability, and recovery behaviour under operational load.

Integration with existing forensic workflows will ensure procedural compatibility and legal admissibility. Mapping standard custody protocols onto SelectVote transactions will support compliance with evidential regulations.

These studies will transition SelectVote from laboratory validation to deployable forensic infrastructure.

## Figures and Tables

**Table 1:** Consensus Mechanism Performance Comparison.

Metric	SelectVote	Hashgraph	GM-PBFT	DIANA-PBFT	Double-Layer
Communication	$O(n^{1.7})$	$O(n^{2})$	O(nlogn) *	$O(n^{2})$	O(n) ^†^
Finality	<20 ms	50 ms	100 ms	150 ms	50–100 ms
Witness Selection	Deterministic 20%	All nodes	Groups	Reputation	Two-tier
Environmental Comp.	Yes	No	No	No	No
Weight Verification	Yes	No	No	No	No

* Under optimal grouping conditions; ^†^ Data layer only; consensus remains $O(n^{2})$.

**Table 2:** Weight-Based Tampering Detection Thresholds.

Evidence Type	Mass (g)	±2% Precision (g)	Detectable Mod.	Sub-Gram Detection
Memory chip	2	±0.04	>0.04 g	✓
MicroSD card	10	±0.2	>0.2 g	✓
USB flash drive	20	±0.4	>0.4 g	✓
Small IoT device	50	±1.0	>1.0 g	Threshold
Smartphone	150	±3.0	>3 g	
Tablet device	500	±10	>10 g	
Laptop computer	2000	±40	>40 g	

## Data Availability

The raw data supporting the conclusions of this article will be made available by the authors on request.

## Abbreviations

The following abbreviations are used in this manuscript:

BFT	Byzantine Fault Tolerance
DAG	Directed Acyclic Graph
TDAG	Transaction-based Directed Acyclic Graph
PBFT	Practical Byzantine Fault Tolerance
IOTA	Internet of Things Application (Tangle cryptocurrency)
SPECTRE	Serialization of Proof-of-work Events: Confirming Transactions via Recursive Elections
FFDCC	First-Fit Decreasing with Case Constraints
SV-BFT	SelectVote Byzantine Fault Tolerance
AES	Advanced Encryption Standard
ECDSA	Elliptic Curve Digital Signature Algorithm
SHA	Secure Hash Algorithm
API	Application Programming Interface
GDPR	General Data Protection Regulation
CPIA	Criminal Procedure and Investigations Act
NPCC	National Police Chiefs’ Council
RH	Relative Humidity
TPS	Transactions Per Second
kPa	Kilopascals
